# Four Challenges That Global Health Networks Face

**DOI:** 10.15171/ijhpm.2017.14

**Published:** 2017-02-08

**Authors:** Jeremy Shiffman

**Affiliations:** Department of Public Administration and Policy, School of Public Affairs, American University, Washington, DC, USA.

**Keywords:** Global Health Networks, Effectiveness, Framing, Governance, Coalition-Building

## Abstract

Global health networks, webs of individuals and organizations with a shared concern for a particular condition, have proliferated over the past quarter century. They differ in their effectiveness, a factor that may help explain why resource allocations vary across health conditions and do not correspond closely with disease burden. Drawing on findings from recently concluded studies of eight global health networks—addressing alcohol harm, early childhood development (ECD), maternal mortality, neonatal mortality, pneumonia, surgically-treatable conditions, tobacco use, and tuberculosis—I identify four challenges that networks face in generating attention and resources for the conditions that concern them. The first is *problem definition:* generating consensus on what the problem is and how it should be addressed. The second is *positioning:* portraying the issue in ways that inspire external audiences to act. The third is *coalition-building:* forging alliances with these external actors, particularly ones outside the health sector. The fourth is *governance:* establishing institutions to facilitate collective action. Research indicates that global health networks that effectively tackle these challenges are more likely to garner support to address the conditions that concern them. In addition to the effectiveness of networks, I also consider their legitimacy, identifying reasons both to affirm and to question their right to exert power.

## Introduction

Over the past quarter century global health networks have proliferated. Global health networks are webs of individuals and organizations linked by a shared concern to address a condition that affects or potentially affects a sizeable portion of the world’s population.^1^ These now exist for most major health conditions in low- and middle-income countries. Many—sometimes referred to as global health initiatives—are governed by formal institutions. Among the best known are the Global Polio Eradication Initiative and Roll Back Malaria. Others are characterized by informal ties, such as an emerging network concerned with rheumatic heart disease. The spread of these networks represents a transformation in the way global health is governed: from a system largely dominated by hierarchical forms of organization—particularly nation-states and inter-state organizations—to one also characterized by horizontal networking and growing participation of non-state actors. Differences in the effectiveness of these networks may be one reason for the considerable variance that exists in the amount of attention and resources global health conditions receive, variance not well explained by so-called ‘rational’ factors such as burden of disease and the availability and cost-effectiveness of interventions.

I identify four strategic challenges that global health networks commonly face: problem definition, positioning, coalition-building and governance (Figure). I do so by drawing on findings from recently concluded studies of eight global health networks addressing alcohol harm, early childhood development (ECD), maternal mortality, neonatal mortality, pneumonia, surgically-treatable conditions, tobacco use and tuberculosis.^1-13^ These case studies provide evidence that networks that effectively address these challenges increase the likelihood of generating attention and resources for the conditions that concern them.

**Figure:**
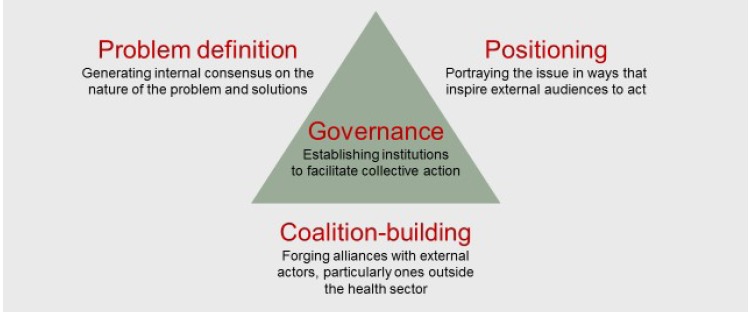
Four Challenges That Global Health Networks Face

## The Four Challenges

The first two challenges, problem definition and positioning, pertain to framing. Framing is a process of constructing meaning that enables individuals to organize experience, to simplify and make sense of the world around them, and to justify and facilitate collective action.^14,15^ *Problem definition* pertains to a challenge internal to the network: how members understand the problem and its solutions. Problems and solutions can be conceptualized in many ways.^16^ For instance, those involved with population and reproductive health policy have disagreed on whether individual rights or social consequences provide the primary rationale for addressing these issues, and on the centrality of family planning provision in this agenda.^17^ Global health networks often become embroiled in conflict over problem specification and solutions, hampering their ability to act collectively.

If problem definition is largely an internal framing matter, *positioning* is an external framing concern: how the network portrays the issue to external audiences. Any given issue can be portrayed in multiple ways, and only some may resonate with the external actors whose resources are needed to make progress in addressing a problem. For example, HIV/AIDS has been portrayed as a public health problem, a development issue, a humanitarian crisis and a threat to security.^18-20^ Some positionings resonate more than others, and different positionings appeal to different audiences. Finance ministers, for instance, might be more likely to respond to portrayals that emphasize the economic costs of a health problem than are health ministers, who might pay more attention to ones that focus on public health benefits and losses. The external positionings networks adopt usually mirror the problem definitions they create. The concepts of problem definition and positioning are linked also in that both are grounded in a social constructionist perspective: issue portrayals are not dictated by a fixed external reality, but rather are constructed by actors concerned with the problem.^16,21-23^

*Coalition-building* pertains to the recruitment of allies beyond core proponents. Many global health networks are insular: they consist largely of individuals and organizations within the health sector and with a specific focus on the issue. Research indicates that those networks that build coalitions that reach beyond like-minded actors and that extend beyond the health sector—a task that necessitates engagement in the politics of the issue, not just its technical dimensions—are more likely to achieve their objectives.^3^

*Governance* pertains to the establishment of institutions to facilitate collective action. Provan and Kenis^24^ identify three primary modes of network governance: (1) shared, where most or all network members interact on a relatively equal basis to make decisions; (2) lead organization, where all major network-level activities and key decisions are coordinated through and by a single participating member; and (3) network administrative organization, where a separate entity is set up specifically to govern the network and its activities. It is not that one mode is better than others: the question is whether the mode is congruent with particular characteristics of the network. For instance, a small network whose members trust one another and agree upon goals may be destroyed if a single individual or organization with a particular agenda comes to dominate it; a large network whose members lack trust in one another and who disagree on goals may need a lead organization to bring about effective collective action.^1^

## The Eight Cases

The eight case studies provide evidence of the influence of problem definition, positioning, coalition-building, and governance decisions on network capacity to generate attention and resources for the issues that concern them. Two of the case studies—on ECD^12^ and surgically-treatable conditions^13^—were conducted as independent studies, motivated by recognition that these issues present a large global burden but receive insufficient attention and resources. Six of the case studies^4-9^ were conducted as part of a larger research project funded by the Bill and Melinda Gates Foundation examining the emergence and effectiveness of global health networks.^2^ In that project the six networks were grouped into three matched pairs: two communicable diseases that affect the respiratory system (tuberculosis and pneumonia); two groups at risk at birth (pregnant women and newborns); and two addictive substances (tobacco and alcohol). Within each pair, despite comparable or lower disease burden, the first issue has received greater policy attention than the second. The project aimed to explain why.

A theoretical framework on the emergence and effectiveness of global health networks,^1^ developed as part of the six case research project, informed analysis of all eight cases. It consists of 10 factors in in three categories: (1) features of the networks and actors that comprise them, including leadership, governance arrangements, network composition and framing strategies; (2) conditions in the global policy environment, including potential allies and opponents, funding availability and global expectations concerning which issues should be prioritized; (3) and characteristics of the issue, including severity, tractability and affected groups. Project researchers began with the presumption that factors in all three of these categories—not just network features—shape policy priority, and examined the role of these factors.

The issues these eight networks address differ in the level of global policy attention they have received ( Table, column 6). Tuberculosis, tobacco control and maternal mortality have received the greatest attention and resources. Priority for neonatal mortality, pneumonia and ECD has been moderate. Attention has been weakest for surgically-treatable conditions and alcohol harm. The networks also vary in their effectiveness in addressing these four challenges, differences that help to explain divergent levels of priority for the issues that concern them ( Table, columns 3,4,5). Networks addressing tuberculosis, tobacco control and maternal mortality have fared best, producing relatively cohesive problem definitions and positionings of the issue, forging broad and stable coalitions that extend beyond the health sector, and establishing governance mechanisms that effectively bring together concerned actors. Networks addressing ECD, surgically-treatable conditions, alcohol harm and pneumonia have fared worst, with contested problem definitions, narrow or unstable coalitions, and fragmented governance—although in recent years these networks have progressed in addressing these challenges. A network addressing neonatal mortality stands as an intermediate case, with cohesive problem definition and governance, but difficulties with positioning and a narrow coalition comprised largely of technical actors in the health sector.

**Table:** Network Recent Form, Status of Challenges and Level of Priority for Issue

**Network**	**Most Recent Structure/Organization** ^a^	**Problem Definition and Positioning**	**Coalition-Building**	**Governance**	**Level of Global Priority for Issue** ^b^
Alcohol harm	2000: Global Alcohol Policy Alliance forms, bringing together more than 200 alcohol policy and public health advocates from about 30 countries.	*Contested*: Public health framing competes with individual behavioral and medical framings.	*Narrow*: Largely researchers from high-income countries.	*Fragmented*: Networks grounded in divergent framings largely operate separately from one another.	*Weak*: Non-binding global strategy not adopted until 2010 and to date has had minimal impact on national priority.
ECD	2016: Several forums have emerged that link organizations working on ECD, including the Saving Brains Initiative and an alliance between the World Bank and UNICEF to prioritize ECD.	*Contested*: Disagreement about the boundaries of the field, the time period constituted by early childhood, and priority interventions; this has made it difficult for advancing a case for ECD that political leaders and the public can easily understand.	*Broad but unstable*: Comprised of experts concerned with health, nutrition, education, social welfare and child protection, but ties among them are unsteady.	*Fragmented*: No institution exercises global leadership on the issue, and interests diverge among involved organizations.	*Moderate*: More than 60 countries have adopted ECD policy; six major global declarations since 1990; a programmatic focus in major global institutions (including WHO, World Bank, UNICEF and UNESCO); ECD-related targets included in SDGs.
Maternal mortality	2005: PMNCH forms, although it is only one among multiple institutions that presently connect maternal health actors. As of 2015, PMNCH linked more than 680 organizations.	*Cohesive*: An ethical imperative—a matter of women’s rights and equity—that requires urgent action due to slow progress.	*Broad*: Initially insular, evolves into political coalition linking researchers, advocates and politicians from high- and low-income countries.	*Cohesive*: Although no single global guiding institution, involved individuals and organizations work largely in tandem, linked by framing of issue as an ethical imperative.	*Strong*: 2010 UN-organized plan with heavy maternal health component; $3.0 billion in donor funding in 2014 alone; maternal mortality prominent in MDGs and SDGs.
Neonatal mortality	2000: Newborn survival program founded at the Save the Children USA (SNL). Since then SNL and small, informal group of researchers and program officers from multiple organizations constitute network’s core and exercise global leadership on the issue.	*Cohesive but not yet adequate*: General agreement within community on problem definition. Still searching for positioning that large numbers of political leaders find compelling.	*Narrow but broadening*: Tight core of health-oriented professionals; expansion beyond health sector historically has been slow but is growing in SDG era.	*Cohesive*: An informal network of health-oriented professionals exercises strong leadership, bringing together multiple organizations.	*Moderate but growing*: As of 2010 only $613 million in donor non-research disbursements across time for the issue; however, in 2014 a global newborn action plan is produced; also inclusion of neonatal mortality reduction target in SDGs is indicative of growing priority.
Pneumonia	2003: Influential actors begin to rebuild a dormant network around a broader identity encompassing a larger spectrum of interventions, including vaccines.	*Contested*: Forceful positioning as ‘leading killer of children,’ but historically disagreement over whether it should be a stand-alone issue or integrated into child survival.	*Narrow and unstable*: Network emerges, dissolves then reappears—a function of shifting ties with broader child survival initiatives and internal differences over interventions.	*Fragmented*: No central guiding forum or institution that brings together primary organizations.	*Moderate*: In 2013, pneumococcal vaccine policies in 192 countries but other interventions lagging.
Surgically-treatable conditions	2015: *Lancet* Commission on Global Surgery and Global Alliance for Surgical, Obstetric, Trauma, and Anesthesia Care (G4 Alliance) attempt to unify surgeons and others to address surgically-treatable conditions.	*Contested but growing cohesion*: Agreement on problem definition—a lack of surgical services in low-income settings—but no widespread agreement yet on strategies to address problem or on public positioning of the issue.	*Narrow*: Comprised primarily of surgeons and anesthesiologists, most from high-income countries.	*Fragmented but growing cohesion*: Lancet Commission, G4 Alliance and WHO helping to forge ties among involved actors, and serving as global convening forums.	*Weak but growing*: No major global health donor provides more than minimal resources for surgery and the MDGs/SDGs do not mention it; however, 2015 passage of World Health Assembly Resolution on Strengthening Emergency and Essential Surgical Care and Anesthesia as a Component of Universal Health Coverage.
Tobacco control	1999: FCA forms as formal coalition of NGOs around global tobacco control treaty; over past decade, expansion and decentralization of network including new funding partner networks, regional networks, and national-level coalitions.	*Cohesive*: A public health threat, with industry as the vector of disease.	*Relatively broad*: Researchers and advocates from high- and low-income countries.	*Largely cohesive*: Multiple networks and organizations work largely in tandem, unified by framing.	*Strong*: Legally binding treaty (FCTC) enacted in 2003 that has compelled nation-states to carry out tobacco control measures.
Tuberculosis	2001: Coalition is formalized in the form of the Stop TB Partnership, which as of 2012 encompassed approximately 1600 individuals and organizations.	*Relatively cohesive*: A social threat, with DOTS as core strategy to address the disease (although some disagreement on DOTS’ efficacy).	*Broad*: Researchers, advocates and political leaders from high- and low-income countries.	*Largely cohesive*: Stop TB Partnership serves as primary global guiding institution, linking major individuals and organizations.	*Strong*: In 2014 alone, $1.4 billion in donor funding and primary strategy, DOTS, implemented in 180 countries.

Abbreviations: PMNCH, Partnership for Maternal, Newborn and Child Health; FCA, Framework Convention Alliance; NGOs, non-governmental organizations; DOTS, directly observed treatment short-course; UNICEF, The United Nations Children’s Fund; SDG, Sustainable Development Goal; WHO, World Health Organization; UNESCO, United Nations Educational, Scientific and Cultural Organization; MDG, Millennium Development Goal; UN, United Nations; ECD, early childhood development; TB, tuberculosis; FCTC, Framework Convention on Tobacco Control. ^a^ Sources of information on network recent form and on problem definition, positioning, coalition-building and governance: alcohol harm^9^; early childhood development^12^; maternal mortality^6^; neonatal mortality^7^; pneumonia^5^; surgically-treatable conditions^13^; tobacco control^8^; tuberculosis.^4^ ^b^ Sources for information on priority: alcohol harm^9^; early childhood development^12^; maternal mortality^25^; neonatal mortality^7,26^; pneumonia^27^; surgically-treatable conditions^13^; tobacco control^8^; tuberculosis.^25,28^

## More Effective Networks: Tuberculosis, Tobacco Control, Maternal Survival

A perception of tuberculosis as a social threat and the existence of a medical specialty led to the formation of institutions to address the disease as early as the mid-1800s, a process that continued through the 20th century.^4^ These institutions in turn shaped the formation in the 1990s of a strong coalition linking researchers, donors, advocates and political leaders, most of whom embraced a common problem definition of tuberculosis as a global public health emergency and DOTS (directly observed treatment short-course) as a strategy to address the disease.^4,28^ In 2001, this coalition was formalized in the form of the Stop TB Partnership, offering a governance structure to guide global action on the issue, with the World Health Organization (WHO) as Secretariat. The Partnership facilitated network growth and the adoption by national governments of DOTS. As of 2012, Stop TB Partnership individual and organizational membership had reached approximately 1600, and the number of advocacy non-governmental organizations (NGOs) and local organizations signing onto a global plan to address the disease continues to grow.^4^ Research by TB network members has informed country strategic plans, particularly in the 22 highest burden countries.^29^ The network’s strength enabled it to take advantage of opportunities for generating attention and resources—including the Millennium Development Goals (MDGs), the creation of the Global Fund to Fight AIDS, Tuberculosis and Malaria and HIV-TB co-infection—and to influence cross-national policy adoption and the scaling-up of interventions. Not all has been smooth for TB advocacy, however: in recent years, the Partnership has struggled to adapt to address the changing nature of the epidemic, including the emergence of multi-drug resistant TB.^4^

A tobacco control network, tight-knit and with strict entry requirements (eg, no contact with industry), has evolved into a strong political coalition linking researchers and activists. Coalition members share a common problem definition and have advanced a cohesive public positioning of the issue: tobacco use as a public health threat, the industry as the vector of disease and governments as having an obligation to enact anti-tobacco legislation.^8,10^ The current form of the network had its origins in the 1990s, when tobacco control proponents from around the world augmented their activities surrounding negotiations of a global treaty on tobacco control. During the treaty negotiations, proponents brought together dozens of NGOs working on the issue, leading to the creation of a formal network organization in 1999—the Framework Convention Alliance (FCA)—that has exercised effective governance surrounding the issue.^8^ Since the adoption of the 2003 Framework Convention on Tobacco Control (FCTC), the first treaty negotiated under the auspices of the WHO, network members have made deliberate efforts to expand beyond an original core. They have brought in and built regional and national networks and extensive civil society involvement,^8^ leading to an expansion in formal network membership of the FCA from 60 organizations in 1999 to approximately 500 presently. By pushing for and monitoring country compliance with the FCTC, tobacco network members helped to marginalize the tobacco industry and to facilitate a doubling in the number of people protected by comprehensive smoke-free laws—to 787 million—between 2008 and 2010.^8,30^ They have also influenced policy on other issues such as pictorial health warnings and advertising bans.

Proponents concerned with addressing maternal mortality have had a challenging history.^6^ They launched a safe motherhood initiative in 1987, but soon thereafter became embroiled in internal disputes connected to problem definition, particularly pertaining to intervention strategy: the relative importance of skilled attendance at birth versus emergency obstetric care.^20^ In addition, for 15 years following the launch of the initiative, the network’s composition was limited largely to technical actors from Northern agencies.^6,20^ Moreover, the network was unable to attract many women’s rights advocates—seemingly natural allies—who objected to the use of the initial term for the initiative, ‘safe motherhood,’ because of its focus on the reproductive role of women. These developments hampered the network’s ability to convince policy-makers to act on the problem.

Over the past decade, however, maternal survival has garnered greater political support and resources.^6^ In the late 2000s, although not all of these represented new pledges, proponents helped to draw an estimated $40 billion in commitments from 127 stakeholders for the Global Strategy for Women’s and Children’s Health. One reason was slow progress on the maternal survival MDG and growing expectations that governments prioritize women’s rights and health, which put pressure on political leaders to act.^6^ Another is that after two decades of disagreements on interventions, in the mid-2000s prominent maternal survival proponents coalesced around a common problem definition emphasizing emergency obstetric care, skilled attendance at birth and access to comprehensive reproductive health services, including family planning.^6,20^ In addition, they advanced a more effective positioning of the issue, emphasizing its social equity and women’s rights dimensions, enabling them to build a broader coalition for the issue that included the *United Nations* (UN) Secretary-General and heads of state from low and high-income countries.^6^

## Networks That Are Struggling: Early Childhood Development, Surgery, Alcohol Harm, Pneumonia

The inter-sectoral nature of early child development has posed opportunities and challenges for the network advancing the issue. The emergent network is broad—an advantage for coalition-building—linking individuals and organizations from several sectors, including health, nutrition, education, social welfare, and social protection.^12^ However, network members disagree on several fundamental issues pertaining to the definition of the problem and its solutions—including the boundaries of the field, the period constituted by early childhood, and priority interventions—making this coalition unstable, and presenting difficulties for developing a public positioning of the issue that could generate political support. In addition, disagreements among involved actors and competition for scarce resources among sectors has precluded the establishment of effective governance arrangements at global and national levels. One point of governance disagreement concerns integration: whether individual sectoral strategies work best, or integrated programs in which health, nutrition, education, and other services are jointly funded, managed, implemented, and evaluated as seamless services. Despite difficulties, ECD proponents have made advances in recent years on the establishment of global forums linking actors addressing the issue, the adoption of global resolutions, the production of research making a strong investment case for ECD, and the development of indicators.

A nascent global surgery network also faces problems.^13^ The coalition is narrow, comprised almost exclusively of surgeons and anesthesiologists, most from high-income settings. They have made little effort to harness the voices of patients at the grassroots level, and existing civil society institutions and forums that promote global surgery are largely limited to professionals. With respect to problem definition, although there is widespread consensus within the community that surgical capacity in low- and middle-income countries is grossly neglected, there are large differences over how to address this problem. There is disagreement even on the basic issue of how to define surgery and surgical care. As with the ECD community, these difficulties with problem definition have hampered positioning efforts, a challenge compounded by widespread public misperception that the provision of surgical services is costly and by the preference of global health funders for disease-specific initiatives over horizontal causes such as surgery provision. The community is also struggling to build effective global governance arrangements, although the recent establishment of a *Lancet* commission on global surgery is helping to build ties among proponents, as well as to address the difficulties with problem definition and positioning. A notable recent success of the community is the passage of a 2015 World Health Assembly resolution on surgical care and anesthesia as a component of universal health coverage.

A global alcohol harm network has struggled due to narrow composition, disagreements with other groups on problem definition, and fragmented global governance of the issue.^9,10^ The network consists predominantly of researchers from North American and European institutions linked by an understanding of alcohol harm as a threat to public health. Its members have not engaged extensively in coalition-building activities. They have faced other groups that view the issue not as a public health but as an individual behavioral or medical problem. The failure of prohibition stands as the backdrop to these competing problem definitions and to different approaches to addressing alcohol harm. Although 66 WHO member states had written national alcohol policies as of 2012,^31^ few countries have strong programs to address alcohol harm.^9^ There is some momentum for the issue, however: the network contributed to the development and passage of a Global Strategy to Reduce the Harmful Use of Alcohol, adopted by the World Health Assembly in 2010.

A pneumonia network, consisting predominantly of researchers and program officers in the health sector, has been slow to coalesce and only emerged as a consequential actor in global health in the past several years.^5^ Several factors have stood behind this slow coalescence. Pneumonia historically has rarely been understood as a social threat—a problem connected to public perception and positioning. Unlike tuberculosis, it never inspired the formation of a medical specialty dedicated to address it, hampering the development of effective governance at the global level. Disagreements over intervention strategy, while less stark now, fragmented the community of individuals concerned with the disease, making it difficult to generate a cohesive problem definition. Perhaps most fundamentally, efforts to address the disease have had an uncertain relationship with broader child survival initiatives, at times operating separately, at other times subsumed under these efforts. This uncertain relationship has created difficulties for establishing a cohesive definition of the problem, for developing a strong public positioning of the issue, and for building effective global governance mechanisms. These difficulties have meant that while global efforts to address pneumonia have proceeded, the network has only been a secondary force in shaping attention to the disease, in promoting national policy adoption and in facilitating mortality decline.^5^

## An Intermediate Case: Newborn Survival

A newborn survival network represents an intermediate case.^7^ It has been more effective in generating attention to its concern than networks addressing ECD, surgically-treatable conditions, alcohol harm and pneumonia. However, to date it has not been as effective as a comparable network—maternal survival—which also targets a group at-risk at birth.^11^

Emerging in the early 2000s, the network has been remarkably cohesive, guided by a small, informal group of committed health professionals and the health-oriented agencies they work for. While there have been some internal disagreements on problem definition pertaining to intervention strategy, these have been minor and managed largely without causing fragmentation in the community—a contrast to the early years of the maternal survival initiative. Moreover, the network has cohered around a sharp focus on the specific problem of the survival of babies under one month of age, and a sustained consensus that initiatives ought not to stand alone but rather be integrated with broader child and maternal survival efforts.

While governance and problem definition have been network strengths, positioning and coalition-building have presented challenges. Network members have focused largely if not exclusively on technical dimensions of the issue. While they have advanced arguments for attention to the issue (especially its rising share of child mortality and its centrality to achieving global child survival goals), they have yet to discover a positioning that provides a sense of urgency and that national political leaders have found sufficiently compelling to justify the provision of extensive public resources. Moreover, while expanding to some degree, the composition of the network’s core has changed little since its emergence in the 2000s. In recent years, however, there has been progress: network members have mobilized parent groups on preterm birth, secured passage of a global newborn action plan, influenced the adoption of national plans in countries with high neonatal mortality including India and Nigeria, and helped to secure a neonatal mortality reduction target in the Sustainable Development Goals (SDGs). It should be noted that the neonatal mortality network emerged fifteen years after a maternal mortality network; in the SDG era, it may see the success that the maternal mortality network did in the MDG era.

## The Question of Network Legitimacy

The proliferation of networks raises a question about their legitimacy: by what authority do they exert power? Democratic theorists offer strong reasons for not taking legitimacy for granted, contending that the right to exert power is contingent not just on performance—what they term output legitimacy—but also fair process, inclusive deliberation and transparency—or input legitimacy.^32-34^

On output and input legitimacy grounds, there are several reasons to consider global health networks legitimate actors in global health governance.^3^ First, they raise attention to and resources for high burden health conditions that national governments might otherwise have neglected or failed to address adequately. Second, they bring considerable expertise to bear on these problems; in their absence, we would know much less about their scope and how to address them. Third, they add new voices—including some from civil society—to policy processes that might otherwise have been dominated by national governments and international organizations. However, there are also reasons to raise questions.^3^ First, elites from Northern institutions have controlled many of these networks; in the majority, Southern institutions have had limited representation and even more so for citizens of Southern countries—the often marginalized individuals most affected by the problems that these networks seek to address. Second, these networks in some instances have contributed to the fragmentation of global and national health governance, hampering the creation of cohesive global health strategies and strong national health systems.

The larger issue is the place of these networks in the governance of global and national health: to what extent do the deficiencies of international organizations and national governments in addressing pressing health problems justify their existence; to what extent do they exert power without legitimate authority? There may be some truth in both perspectives.

## Conclusion

Global health network effectiveness is of course a function of much more than member decisions on problem definition, positioning, coalition-building and governance. Factors such as the availability of cost-effective interventions, disease burden, crises, the fears and interests of powerful nation-states, the inclusion of conditions in global development goals such as the SDGs, and the availability of donor funding also influence network effectiveness, as well as the amount of attention and resources conditions receive. However, considerable research indicates that the way networks manage these four challenges has substantial influence on the likelihood that they achieve their objectives. It makes sense, therefore, for networks to address these challenges explicitly rather than to leave decisions on problem definition, positioning, coalition-building and governance to forces outside their control.

## Acknowledgements

I would like to thank Stephanie Smith and Yusra Shawar for their thoughtful and valuable comments on drafts of this essay. I am grateful also for the research support for the case studies provided by the Bill and Melinda Gates Foundation, and the US Fund for UNICEF through funds from the Conrad N. Hilton Foundation.

## Ethical issues

Not applicable.

## Competing interests

Author declares that he has no competing interests.

## Author’s contribution

JS is the single author of the paper.
